# Evaluation of Gastric Emptying Time of a Rice-Based Meal Using Serial Sonography

**DOI:** 10.1155/2019/5917085

**Published:** 2019-10-28

**Authors:** Eun-Ah Cho, Mi Sung Kim, Yun Byeong Cha, Mi-Suk Lee, Taejong Song

**Affiliations:** ^1^Department of Anesthesiology and Pain Medicine, Kangbuk Samsung Hospital, Sungkyunkwan University School of Medicine, Seoul, Republic of Korea; ^2^Department of Radiology, Kangbuk Samsung Hospital, Sungkyunkwan University School of Medicine, Seoul, Republic of Korea; ^3^Department of Obstetrics & Gynecology, Kangbuk Samsung Hospital, Sungkyunkwan University School of Medicine, Seoul, Republic of Korea

## Abstract

The aim of this prospective study was to evaluate the gastric emptying time of a rice-based meal by serial ultrasonography of the stomach. After baseline ultrasonographic assessment of ten fasted healthy volunteers, volunteers ingested standardized 420 g, 536 kcal rice-based meal (bibimbap), and serial evaluations were performed every hour until the stomach became empty. At baseline, all the participants had an empty stomach. The average time of complete gastric emptying of the rice-based meal was 5.8 ± 0.8 h (95% confidence interval (CI), 5.0 h to 6.5 h). Since the first postintake cross-sectional area (CSA) measurement, a decrease was observed, and CSA was maintained until postprandial 3–4 h (*P* > 0.05). It declined rapidly 4 h after meal intake (*P*=0.031), reaching the nadir at approximately 6 h after meal intake. The gastric CSA and hunger score showed a positive correlation (correlation *r* = 0.616, *P* < 0). The rice-based meal is emptied after 5.8 ± 0.8 h on average in healthy volunteers. Based on our results, 6.5 h (upper limit of CI) of fasting after the ingestion of a rice-based meal would be a safe preoperative fasting time, and this is in accordance with the current guidelines for preoperative fasting.

## 1. Introduction

Pulmonary aspiration of gastric contents during sedation or general anesthesia is rare [1]. However, if it occurs, serious complications and even death can be a consequence [2, 3]. Gastric volume is thought to be one of the important risk factors of pulmonary aspiration, which can be minimized by fasting [4]. Therefore, fasting before sedation or administration of general anesthesia has long been considered as a routine policy to reduce pulmonary aspiration [5, 6].

As a conventional fasting policy, nil per mouth (*nulla per os*, NPO) from midnight or 8 h of fasting is implemented by most medical centers, according to the recommendation of the American Society of Anesthesiologists (ASA) guidelines [6]. Current ASA guidelines recommend a minimum fasting period of 6 h if the meal is light (toast or sandwich) and 8 h if the meal includes fried or fatty foods [5]. In the era of evidence-based medicine, however, it is not sufficient to accommodate these fasting recommendations, which are derived from survey opinions solicited by 470 active members of the ASA [5, 7]. Furthermore, these guidelines were framed according to the western style meals such as toasts or sandwiches.

Recent studies have shown that real-time ultrasonography can provide reliable information about the nature (clear fluid, solid, or none) and volume of gastric content; it is a noninvasive, inexpensive, and safe method [4, 8–10]. It is currently accepted that real-time gastric ultrasonography of antral cross-sectional area (CSA) allows the accurate determination of gastric emptying time, similar to scintigraphy or barium X-ray of the stomach [11, 12]. The ultrasonographic measurement of the antral CSA is especially used for the assessment of gastric emptying before the administration of obstetrical and pediatric anesthesia [13–16]. However, for rice-based meals, there have been no previous studies regarding the gastric emptying time or guidelines concerning fasting period. Therefore, the objective of this prospective observational study was to evaluate gastric emptying time of a rice-based meal (Asian foods) by evaluating the stomach with serial ultrasonography.

## 2. Methods

### 2.1. Participants

The study was approved by the Institutional Review Board of Kangbuk Samsung Hospital, Seoul, Republic of Korea (Approval number: KBSMC 2018-08-018), and all data were collected prospectively. The study was conducted in compliance with the Korean Good Clinical Practice guidelines and the Declaration of Helsinki. Healthy volunteers who visited our medical center for medical checkups or as patient caregivers were asked to participate in this study. Written informed consents were obtained from all volunteers.

The inclusion criteria were as follows: age over 18 years, class I or II physical status according to ASA, and body mass index (BMI) between 18.5 and 29.9 kg·m^−2^. Exclusion criteria were as follows: history of upper abdomen surgery and gastrointestinal diseases such as hiatal hernia and gastroesophageal reflux, diabetes mellitus, chronic kidney disease, or pregnancy.

### 2.2. Gastric Sonography

To minimize intra- and interrater variability, gastric sonography was assessed by the two independent investigators, three consecutive measurements in each investigator, in all healthy volunteers. A certified radiologist (M.S. Kim) with 18 years of experience in abdominal ultrasonography (approximately 850 examinations per year) performed all the ultrasonographic assessments. An anesthesiologist (E. A. Cho) previously received didactic teaching of a 30 min of lecture on the ultrasonographic gastric assessment and trained in hands-on workshop and a live demonstration of ultrasonographic assessment performed second assessment of gastric ultrasonography.

The volunteers were permitted their last oral intake before 10 p.m. and were asked not to drink or eat from 10 p.m. until the baseline assessment of gastric sonography. The baseline measurement of the empty gastric antrum was conducted at 8 a.m. on the following day. Then, all volunteers ate usual quantities of the standard solid meal. The standard solid meal was a 420 g prepackaged rice-based meal (bibimbap), which provides 536 kcal of energy and contains warm white rice topped with the following ingredients: fried egg, seasoned vegetables, sliced meat, chili pepper paste, seasoned mushrooms, and sesame oil. The meal intake was measured by subtracting the weight of meal after eating from that before eating. Postprandial assessment of the gastric antrum was conducted 2 h after the meal intake and after every 1 h thereafter until the gastric antrum was empty based on the ultrasonographic assessment. The food intake index (g/kg), defined by the intake of rice-based meal per body weight, was also measured.

The gastric antrum was examined in the right lateral decubitus position. The gastric antrum was measured using a curvilinear low-frequency C2-8 convex transducer (2–8 MHz) of an ultrasonography machine (MySono U6, Samsung Medicine, Seoul, Republic of Korea) at standard abdominal settings. The gastric antrum was identified at the epigastrium in a parasagittal plane using internal landmarks: left lobe of the liver placing anterior to the gastric antrum, and abdominal aorta and pancreas placing posterior to the gastric antrum, as described in the previous study [8]. The CSA of antrum was calculated using the formula of area of an ellipse: CSA = (LD × SD × *π*)/4 (LD = longest diameter; SD = shortest diameter) [8] and using the automatic ellipsoid tracing tool of sonographic machine. The LD and SD were measured such that the full thickness of the gastric layer from serosa to serosa is included. Serosa was identified as a hyperechoic layer of the outermost surface of the stomach. CSA was measured between peristaltic contractions; three measurements were recorded per session.

### 2.3. Outcome Measures

**Figure 1:**
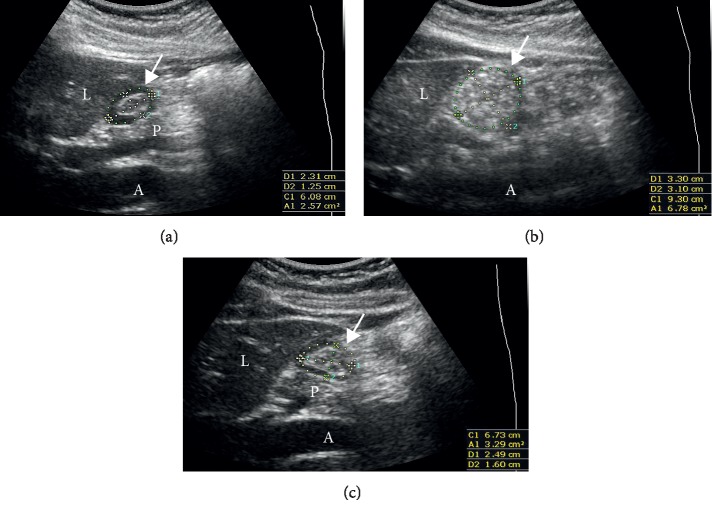
Representative ultrasonographic images of cross-sectional area of the gastric antrum (arrow) in the parasagittal plane: (a) at the time of baseline measurement, indicating fasting stomach, (b) 2 h after the intake of the rice-based meal, and (c) 6 h after the intake of the rice-based meal, indicating empty stomach. A = aorta; L = liver; P = pancreas.

The primary outcome measure was the gastric emptying time determined as the time interval between the last oral intake and gastric emptying detected by serial ultrasonographic assessment of the gastric antrum. The stomach is considered empty when the gastric antrum appears flat with juxtaposed anterior and posterior antral walls, that is, the so-called “target” or “‘bull's eye” pattern (Figure 1) [8]. The examiner was unaware of the quantity of meal intake.

The secondary outcome measure was the degree of hunger. The degree of hunger of the volunteers was self-reported on a hunger scale graded from 1 to 10, where 1 represents beyond hungry and 10 represents beyond full stomach; this scale was adopted from the MIT Medical [17]. The data on hunger score were obtained before every ultrasound measurement. The examiner was also blinded to the degree of hunger.

### 2.4. Statistical Analysis

The sample size was determined with reference to the primary outcome measure, that is, the gastric emptying time. Data from a pilot study (authors' unpublished data) showed that the emptying time was 6.75 ± 1.50 h. Therefore, with a type I error of 0.05, a power of 80%, a margin of error for 1 h of emptying time, and a dropout rate of 10%, the estimated sample size required was 10 participants.

SPSS software 20.0 (SPSS, Inc., Chicago, IL, USA) was used for the statistical analysis. For continuous variables, data are represented as mean ± standard deviation (SD) or median (interquartile range [IQR]) after verifying the normal distribution of the data. For categorical variables, data are presented as frequency (percent). Mann–Whitney tests were used for the comparison of CSA and hunger score between each time point and baseline (or previous time point) measurement. A value of *P* < 0.05 was considered statistically significant.

## 3. Results

The study was conducted from October 2018 to December 2018. Among the 14 persons invited to participate in the study, two declined to participate and two were ineligible because of high (>30 kg·m^−2^) or low BMI (<18.5 kg·m^−2^). Thus, 10 heathy volunteers were enrolled, and they underwent serial ultrasound measurement for gastric emptying; however, one volunteer was further excluded from the analysis because we were unable to localize the antrum at the time of the baseline sonographic measurement.

The baseline characteristics of the study population are summarized in Table 1. The mean age and BMI were 31.4 ± 3.4 years and 22.6 ± 2.5 kg·m^−2^, respectively. The mean weight of the rice-based meal was 321.7 ± 101.0 g, and the mean energy provided was 410.5 ± 128.9 kcal.

The ultrasound measurements of CSA of two investigators are demonstrated in the Supplementary [Supplementary-material supplementary-material-1]. In addition, gastric emptying time measured in two investigators are shown in the Supplementary [Supplementary-material supplementary-material-1]. Gastric emptying time was not different between the two investigators. The ultrasound measurements of the radiologist were used as representative values at each time point and were used for the following analyses.

Table 2 shows the numbers of volunteers with empty stomach, CSA of the gastric antrum, and hunger score at each evaluation time point. The stomach was completely empty in all volunteers (100%) at baseline measurement. The hourly sonographic measurement was carried out after the intake of the standard rice-based meal until the examiner considered the gastric antrum to be empty. Among the 9 volunteers, cumulative numbers of volunteers who underwent postprandial assessment with empty stomach were 0 (0%) after 4 h, 4 (44%) after 5 h, 7 (78%) after 6 h, and 9 (100%) after 7 h. The baseline CSA evaluated using the formula of area of an ellipse was 5.0 ± 1.5 cm^2^ and that evaluated using the automatic tracing tool of sonographic machine was 5.2 ± 1.4 cm^2^. The postprandial CSAs evaluated using the formula were 11.6 ± 3.7 cm^2^, 11.5 ± 2.7 cm^2^, 10.2 ± 2.4 cm^2^, 7.2 ± 2.9 cm^2^, 5.5 ± 1.5 cm^2^, and 6.1 ± 4.6 cm^2^ after 2, 3, 4, 5, 6, and 7 h, respectively. The baseline hunger score was 3.6 ± 0.8, and the postprandial hunger scores were 5.5 ± 0.5, 5.1 ± 0.6, 4.4 ± 0.5, 4.2 ± 0.8, 3.5 ± 0.8, and 3.3 ± 0.6 after 2, 3, 4, 5, 6, and 7 h, respectively.

The average time required for the complete gastric emptying of the rice-based meal in all the volunteers was 5.8 ± 0.8 h (95% confidence interval [CI], 5.0 h to 6.5 h) (Table 3). The stomachs of male and female volunteers were emptied after 5.3 ± 0.6 h and 6.0 ± 0.9 h, respectively. The emptying time of the normal weight volunteers was 5.9 ± 0.9 h, while that of the overweight volunteers was 5.5 ± 0.7 h. Gastric emptying time did not significantly differ according to sex, BMI, and dietary intake quantity (all, *P* > 0.05).

**Figure 2:**
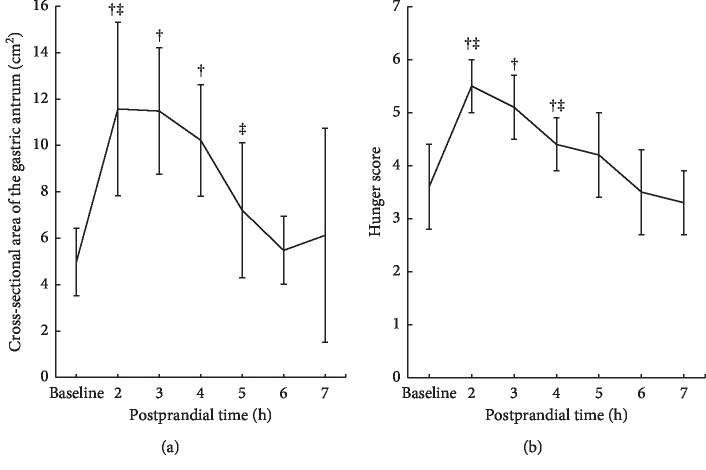
(a) Change in the antral CSA after the ingestion of the rice-based meal over time in 9 healthy volunteers. †The difference between CSA at each time point and baseline measurement was statistically significant at *P* <0.05. ‡The difference between CSA at each time point and the previous measurement was statistically significant at *P* <0.05. (b) Change in hunger score over time after the intake of the rice-based meal. †The difference between hunger scores at each time point and baseline measurement was statistically significant at *P* <0.05. ‡The difference between hunger score at each time point and the previous measurement was statistically significant at *P* <0.05.

**Figure 3:**
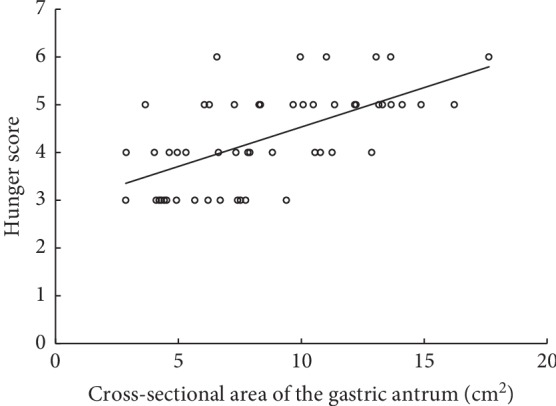
Scatter plot showing the relationship between gastric CSA and hunger score (correlation *r* = 0.616, *P* < 0.001).

Figure 2(a) shows serial changes in the antral CSA over time after the ingestion of the rice-based meal. Since the first postintake cross-sectional area (CSA) measurement, a decrease was observed, and CSA was maintained until 4 h of fasting (*P* > 0.05). It declined rapidly after 4 h of fasting (*P*=0.031), reaching the nadir after approximately 6 h of fasting. Compared with the baseline measurement, the postprandial antral CSA was higher after 2, 3, and 4 h (all, *P* < 0.05), suggesting that the stomach was unemptied; however, it remained similar after 5, 6, and 7 h (all, *P* > 0.05). Additionally, the postprandial hunger score was the highest after 2 h, which gradually decreased over time, which became rapid between 3 and 4 h (*P*=0.024), as displayed in Figure 2(b). The hunger score was significantly higher postprandially after 2, 3, and 4 h than at baseline (all, *P* < 0.05). This returned to the baseline postprandially after approximately 5 h (all, *P*=0.094). The scatter plot representing the relationship between the gastric CSA and hunger score is illustrated in Figure 3. The gastric CSA and hunger score showed a positive correlation (correlation *r* = 0.616, *P* < 0.001).

## 4. Discussion

We conducted this prospective study to investigate the gastric emptying time of a rice-based meal by evaluating the stomach using serial ultrasonography. The main finding of this study was that the average gastric emptying time was 5.8 ± 0.8 h (95% CI, 5.0 h to 6.5 h) for the rice-based meal. We also confirmed that the ultrasonographic measurement of the antral CSA is a reliable test for assessing the gastric emptying in general population. To the best of our knowledge, this is the first study that elucidated the gastric emptying time of rice-based meals. We believe that the present study provides valuable information, which could aid clinicians in determining guidelines for fasting period when patients consume rice-based meals before clinical procedures. In contrast with the minimum fasting period according to ASA guidelines, which is 6 h for a light meal (toast or sandwich) and 8 h for a heavy meal (fried or fatty foods), the fasting time after an Asian meal (rice-based meal) should be at least 6.5 h.

In this study, the average time required to empty the Asian food (rice-based meal) was 5.8 ± 0.8 h (95% CI, 5.0 to 6.5 h). Carmona et al. carried out a prospective study involving 17 healthy volunteers who had ingested a standard 145 g, 355 kcal meat sandwich. They reported that the mean gastric emptying time was 4.5 ± 0.9 h [18]. Using real-time gastric sonography, Bolondi et al. described that the gastric emptying time in general populations was 4.1 ± 0.7 h after the ingestion of a standard 800 kcal Italian meal containing pasta with tomato sauce, meat with vegetables, bread, and water [12]. Beck et al. investigated the gastric emptying time of a standardized light breakfast (one slice of buttered toast with jam or chocolate spread per 10 kg, and clear fluid given without any restriction on volume) in 22 healthy children and reported that the mean gastric emptying time was 3.5 h [19]. Notghi and Hansrod studied gastric emptying time of various western foods using scintigraphy and reported that emptying of egg-based meals, mashed potatoes, and porridge required 90–120 min, 30–60 min, and 30–100 min, respectively [20]. In summary, gastric emptying time seems to be slightly longer for Asian foods than for the western style foods. We thought that this is because of cooking style difference. Unlike the western foods, Asian meals contain a wide variety of ingredients and seasonings.

Our study shows that the ultrasonographic measurement of the antral CSA is a reliable test for the assessment of gastric emptying in general population. Previous studies demonstrated that scintigraphy is a gold standard for measuring the gastric emptying time [11]. However, scintigraphy is accompanied by the risk of radiation exposure. Ultrasonography is a safe, noninvasive, cost-effective method. In addition, ultrasonography is a valid alternative to scintigraphy with low inter-and intra-observer variability [21]. Moreover, the gastric antrum can be identified easily by ultrasonography in most patients, with a relatively short learning curve [21, 22]. Recently, many researchers have measured gastric emptying time of various foods, including liquid and solid foods, using ultrasonography and suggested that the results were well correlated with those of the gold standard method [23]. Therefore, we chose ultrasonography as a method for measuring gastric emptying time in the present study.

Hunger is the one of the main complaints of the patients who fasted ahead of clinical procedures. Various factors, such as emotions, hormones, gastric volume, and the ambient temperatures, affect the feeling of hunger [24]. In our study, hunger score showed a positive correlation with the antral CSA. However, the volunteers felt hungry 1 h before the gastric emptying time assessed by ultrasonography. This observation is consistent with that of the previous studies, which suggested that the hunger score does not exactly predict the gastric volume [24, 25]. Weiji et al. evaluated the predictive value of the hunger score on gastric emptying after oral intake of 8 mL·kg^−1^ of carbohydrate solution in 29 healthy volunteers [24]. They measured the gastric residual volume using magnetic resonance imaging (MRI) and assessed the subjective feeling of hunger using the numerical rating scale (NRS), with 0 representing the feeling of least hunger and 10 representing the feeling of worst hunger. They reported that the subjective hunger NRS score cannot accurately predict the gastric residual volume, but it can provide a reference to clinicians for judging the gastric emptying process. In summary, although the hunger score and gastric CSA are revealed to be well correlated, the former may not accurately predict the gastric emptying.

The present study has some limitations. First, the study was carried out in a small sample size. Therefore, our study was underpowered to detect the difference of the gastric emptying time according to sex and BMI. Moreover, even though we estimated the dropout rate of 10% at the study design, the small sample size was not enough to compare further variables and to include a broad spectrum of population. Furthermore, obese (BMI > 30) patients are not included in this study. Our study included participants with BMI between 18.5 and 29.9 kg·m^−2^, for it represents standard population eating rice-based meal of our country. Therefore, we cannot ascertain whether our results can be applied to the obese populations. Further studies encompassing a broad spectrum of populations with larger sample size might be needed to reach a conclusion. Second, the study was conducted on healthy volunteers. Patients undergoing surgeries often have comorbidities that delay gastric emptying. Moreover, in the clinical settings, clear fluid intake is allowed up to 2 hours prior to anesthesia according to the ASA guidelines. However, in this study, clear fluid was not permitted, to compare the pure gastric emptying time of rice-based meal. Because it is believed that solids and liquids are emptied by the different mechanisms (pressure pump vs. peristalsis) [26], clear liquids might not delay the gastric emptying time of a rice-based meal; however, it needs to be validated in further researches. Therefore, clinicians should consider the risks when applying these results in clinical practice. Third, only two investigators performed the ultrasound measurement. Because measurement of CSA might be differed in every repeated measurement, subanalyses using CSA might have been improved when more investigators had performed gastric ultrasound. Finally, bibimbap was used as a representative rice-based meal in our study. We chose bibimbap because it consists of a mixture of a wide variety of ingredients, which is the common feature of Asian rice-based meal. Different foods containing different ingredients that delay the process gastric emptying might reveal different outcomes. Meanwhile, it was interesting to show the correlation of hunger score and the emptiness of the stomach, and to plot these two and demonstrate the correlation with hunger and full stomach. We suggest that further studies incorporating the relationship of hunger score and measurement of gastric ultrasound would be another interesting topic.

In conclusion, the rice-based meal is emptied from the stomach after 5.8 ± 0.8 h (95% CI, 5.0 h to 6.5 h) on an average. Based on our results, 6.5 h (upper limit of CI) after the ingestion of a rice-based meal would be a safe preoperative fasting period, considering the gastric CSA and the current guidelines for preoperative fasting. However, further large prospective trials using various Asian foods or specific populations are needed to obtain more conclusive data.

## Figures and Tables

**Table 1:** Baseline characteristics of healthy volunteers (*n* = 9).

Baseline characteristics	Value
Gender	
Male	3 (33%)
Female	6 (67%)
Age (yr)	31.4 ± 3.4
Height (cm)	165.3 ± 9.4
Weight (kg)	62.4 ± 13.4
BMI (kg·m^−2^)	22.6 ± 2.5
Smoking	
Current	0
Former	2 (22%)
Never	7 (78%)
Alcohol intake	
None	3 (33%)
Social	6 (67%)
Intake of rice-based meal	
Weight (g)	321.7 ± 101.0
Calorie (kcal)	410.5 ± 128.9
Food intake index^a^ (g/kg) according to BMI classification^b^	
Normal weight (*n* = 7)	5.5 ± 1.4
Overweight (*n* = 2)	4.2 ± 0.9

Abbreviation: BMI, body mass index. Data are presented as mean ± standard deviation or frequency (percent). ^a^Intake of rice-based meal per body weight. ^b^BMI is calculated by dividing the weight in kilograms with the square of the height in meters (kg·m^−2^), and it is categorized into four groups according to the World Health Organization (WHO) guideline: underweight (<18.5 kg·m^−2^), normal weight (18.5–24.9 kg·m^−2^), overweight (25–29.9 kg·m^−2^), and obese (≥30 kg·m^−2^).

**Table 2:** Number of volunteers with empty stomach, cross-sectional area (CSA) of the gastric antrum, and hunger scale score at each evaluation time point.

	Baseline (after 10 h fasting)	Time after meal intake (h)					
		2 h	3 h	4 h	5 h	6 h	7 h
Number of volunteers (*n*)^a^	9 (100%)	9 (100%)	9 (100%)	9 (100%)	9 (100%)	5 (56%)	2 (22%)
Number of volunteers with an empty stomach (*n*)^b^	9 (100%)	0	0	0	4 (44%)	7 (78%)	9 (100%)
CSA by formula (cm^2^)^c^	5.0 ± 1.5	11.6 ± 3.7	11.5 ± 2.7	10.2 ± 2.4	7.2 ± 2.9	5.5 ± 1.5	6.1 ± 4.6
CSA by automatic tracing (cm^2^)^d^	5.2 ± 1.4	12.0 ± 3.7	11.8 ± 2.7	10.6 ± 2.3	7.3 ± 3.0	5.6 ± 1.5	6.5 ± 4.7
Hunger score (1–10)^e^	3.6 ± 0.8	5.5 ± 0.5	5.1 ± 0.6	4.4 ± 0.5	4.2 ± 0.8	3.5 ± 0.8	3.3 ± 0.6

^a^The number of volunteers who received gastric sonographic assessment at each time point is presented as number (%). ^b^The cumulative number of volunteers with empty stomach based on gastric sonography at each time point is presented as number (%). ^c^This CSA was calculated using the formula of the area of an ellipse. ^d^This CSA was calculated using the automatic tracing tool of sonographic machine. ^e^The lower the score, the worse the hunger.

**Table 3:** Emptying time of the rice-based meal according to different categories of volunteers.

Different categories of volunteers	Emptying time of the rice-based meal (h)
All volunteers (*n* = 9)	5.8 ± 0.8
	(95% CI, 5.0 to 6.5)
Sex	
Male (*n* = 3)	5.3 ± 0.6
Female (*n* = 6)	6.0 ± 0.9
BMI classification^a^	
Normal weight (*n* = 7)	5.9 ± 0.9
Overweight (*n* = 2)	5.5 ± 0.7
Quantity of dietary intake	
Low than average (*n* = 6)	5.8 ± 0.8
High than average (*n* = 3)	5.7 ± 1.2

Abbreviation: CI, confidence interval. ^a^BMI is calculated by dividing the weight in kilograms with the square of the height in meters (kg·m^−2^), and it is categorized into four groups according to the World Health Organization (WHO) guideline: underweight (<18.5 kg·m^−2^), normal weight (18.5–24.9 kg·m^−2^), overweight (25–29.9 kg·m^−2^), and obese (≥30 kg·m^−2^).

## Data Availability

The data of the captured image of the ultrasound and other measurements are stored in our Dropbox. These data used to support the findings of this study are available from the corresponding author upon request.

## Supplementary Materials

Supplementary Materials Table S1: interrater variability of the two investigators. Table S2: gastric emptying time (hour) measured by the two investigators.
