# Supplementary material for: Evaluation of Gastric Emptying Time of a Rice-Based Meal Using Serial Sonography
Source: Biomed Res Int. 2019 Oct 28;2019:5917085. doi: 10.1155/2019/5917085 (PMC6855072; doi:10.1155/2019/5917085)
Supplement: Supplementary Materials — Table S1: interrater variability of the two investigators. Table S2: gastric emptying time (hour) measured by the two investigators. [file 5917085.f1.pdf]

Table S1. Interrater variability of CAS in two investigators

	Baseline (after 10 h fasting)	Time after meal intake (h)					
		2 h	3 h	4 h	5 h	6 h	7 h
CSA by formula (cm^2)							
Radiologist	5.0 ± 1.5	11.6 ± 3.7	11.5 ± 2.7	10.2 ± 2.4	7.2 ± 2.9	5.5 ± 1.5	6.1 ± 4.6
Anesthesiologist	4.2 ± 1.4	13.9 ± 6.6	10.8 ± 4.2	9.6 ± 3.4	6.7 ± 2.4	4.9 ± 1.2	5.3 ± 2.9
P-value	0.340	0.605	0.666	0.730	0.863	>0.999	>0.999
CSA by automatic tracing (cm^2)							
Radiologist	5.2 ± 1.4	12.0 ± 3.7	11.8 ± 2.7	10.6 ± 2.3	7.3 ± 3.0	5.6 ± 1.5	6.5 ± 4.7
Anesthesiologist	3.9 ± 1.1	12.6 ± 5.2	10.9 ± 3.9	10.0 ± 3.5	6.9 ± 2.6	5.0 ± 1.1	5.1 ± 3.1
P-value	0.063	0.796	0.489	0.796	0.796	>0.999	0.667

Table S2. Gastric emptying time (hour) measured by the two investigators

Vo1lunteer number	Investigator 1 (radiologist)	Investigator 2 (anesthesiologist)
1	6	6
2	7	7
3	6	6
4	7	7
5	5	5
6	5	5
7	5	5
8	6	6
9	5	5
